# A Molecular and Epidemiological Description of a Severe Porcine Reproductive and Respiratory Syndrome Outbreak in a Commercial Swine Production System in Russia

**DOI:** 10.3390/v14020375

**Published:** 2022-02-11

**Authors:** Karyn A. Havas, Dennis N. Makau, Sergei Shapovalov, Ekaterina Tolkova, Kim VanderWaal, Tymofii Tkachyk, Gordon D. Spronk, Brad Heron, Scott A. Dee, Andres Perez

**Affiliations:** 1 Pipestone Research, Pipestone Holdings, Pipestone, MN 56164, USA; gordon.spronk@pipestone.com (G.D.S.); scott.dee@pipestone.com (S.A.D.); 2 Department of Veterinary Population Medicine, College of Veterinary Medicine, University of Minnesota, Saint Paul, MN 55108, USA; dmakau@umn.edu (D.N.M.); kvw@umn.edu (K.V.); aperez@umn.edu (A.P.); 3 Genomics and Molecular Biology Laboratory, Research and Testing Center, Cherkizovo LLC, 107143 Moscow, Russia; s.shapovalov@cherkizovo.com (S.S.); e.tolkova@cherkizovo.com (E.T.); t.tkachik@cherkizovo.com (T.T.); 4 Pork Division, Cherkizovo Group Limited Liability Corporation, 399870 Lipetsk, Russia; b.heron@cherkizovo.com

**Keywords:** viral emergence, viral evolution, viral transmission, swine production, molecular epidemiology

## Abstract

Porcine reproductive and respiratory syndrome (PRRS) is an economically devastating disease of swine in many parts of the world. Porcine reproductive and respiratory syndrome virus (PRRSV) type 1 is endemic in Europe, and prevalence of the subtypes differ spatially. In this study, we investigated a severe PRRS outbreak reported in 30 farms located in eastern Russia that belong to a large swine production company in the region that was also experiencing a pseudorabies outbreak in the system. Data included 28 ORF5 sequences from samples across 18 of the 25 infected sites, reverse transcriptase real-time polymerase chain reaction (RT-qPCR) results from diagnostic testing, reports of clinical signs, and animal movement records. We observed that the outbreak was due to two distinct variants of wildtype PRRSV type 1 subtype 1 with an average genetic distance of 15%. Results suggest that the wildtype PRRSV variants were introduced into the region around 2019, before affecting this production system (i.e., sow farms, nurseries, and finisher farms). Clinical signs did not differ between the variants, but they did differ by stage of pig production. Biosecurity lapses, including movement of animals from infected farms contributed to disease spread.

## 1. Introduction

Porcine reproductive and respiratory syndrome virus (PRRSV) was first isolated in the early 1990s [1,2]. The associated disease, porcine reproductive and respiratory syndrome (PRRS), is a costly disease of swine that presents in sows with abortions, mummified fetuses, stillbirths, and weak offspring; in piglets with respiratory disease and death; and reduced weight gain in growing pigs [3,4,5]. PRRSV is a positive sense single stranded RNA virus and a member of the family Arteriviridae [3,6]. PRRSV was originally classified into North American and European genotypes [7] and is now classified as *Betaarterivirus suid 2* (PRRSV2) and *Betaarterivirus suid 1* (PRRSV1), respectively.

There is further genetic subtyping amongst the PRRSV1 in Eastern Europe and Russia. Subtype 1 (PRRSV1-1) is most closely related to the Lelystad vaccine variant and variants of subtype 1 are considered less pathogenic [8]. In turn, viral variants in subtype 2 (PRRSV1-2) are believed to cause moderate to severe clinical disease. For example, in a study with three-week old pigs, all infected pigs died and the controls did not [9]. Other studies evaluated subtype 3 (PRRSV1-3) against subtype 1 (PRRSV1-1) and found PRRSV1-3 to be more pathogenic based on clinical score and immune response comparisons [8]. In Russia, in addition to PRRSV1 and PRRSV2 infections [10], atypical sequences of PRRSV1-1 and PRRSV1-2 were also reported [9]. A recent study of atypical sequences of PRRSV revealed mild disease outcomes, infected pigs exhibited mild fever, dyspnea, and inappetence, followed by clinical recovery despite evidence of pneumonia during necropsy [9].

This paper evaluates the movement of the PRRSV virus within a complex swine system that was experiencing a prolonged outbreak of PRRS and describes the genetic diversity found within this system using results from conventional diagnostics. Methods and results presented here illustrate the use of novel phylogenetic tools to support outbreak investigations in intensive swine systems using routine samples and sequencing approaches. The intent is to illustrate the patterns of spread of PRRSV in swine operations.

## 2. Materials and Methods

### 2.1. Outbreak Overview

In the winter and spring of 2020, a severe PRRS outbreak occurred in a swine production system owned by the Cherkizovo LLC (Lipetsk, Russia). Prior to this outbreak, the system did not have PRRSV present in the system, and this disease-free designation was based on the weekly and monthly monitoring for PRRSV that occurred at different stages of production across the system. Since the system was free of PRRSV, vaccine had limited use prior to the outbreak, and it was only used to vaccinate young female animals (gilts) as they were moved to breeding sites. The vaccine used was the Porcilis PRRS vaccine produced by Intervet (Boxmeer, Netherlands) that uses the DV strain of PRRSV1 (http://www.porcilis-prrs.com/spc-porcilis-prrs.asp, accessed on 24 January 2022). Increased vaccination occurred in May 2020 in sow sites, nursery sites where weaned pigs were sent to grow, and sites that provided the breeding animals to the system (multiplication). Three sow sites were also revaccinated in September 2020. Despite these health challenges, Cherkizovo LLC was the fourth largest swine production company in Russia in 2020. In 2019, their revenue was 24.5 billion rubles ($334 million) and in 2020, despite the outbreak, it was 26.5 billion rubles ($362 million). To reach these revenues they marketed 284,167 and 301,547 metric tons of pork in 2019 and 2020, respectively (direct communication, Brad Heron, Director of Operation, Cherkizovo LLC., Lipetsk, Russia).

The Cherkizovo system used a multi-site production model whereby different phases of the pig production lifecycle (i.e., farrowing, weaning, growing, and finishing) occur at separate locations [11,12,13]. There were four contiguous oblasts or regions in southwestern Russia that experienced an outbreak: Voronezh, Lipetsk, Tambov, and Penza. These are four larger geographic regions that are part of the 83 administrative units that form the Russian Federation [14]. The index case was diagnosed with PRRS in March 2020 on a finisher site in Tambov; it had ongoing PRRS-related disease events for the duration of the outbreak. In April, PRRSV was also diagnosed on another site in Tambov and in Voronezh. In Voronezh, most of the disease events occurred in April and May. The outbreak was then observed in Lipetsk and in the fall emerged in Tambov (personal communication, Director of Operations of the Infected Swine System, February 2021)

Pig movements occurred regularly throughout the system and across regions as part of normal operations. There was a multiplier site in the Voronezh region that provided gilts to gilt development sites in the other regions. It received a load of gilts that were imported into Russia and quarantined at a site in Lipetsk. After the quarantine ended, the gilts were sent to Voronezh and tested negative for PRRSV, and then five days later had a questionable test result and were eventually found to be PRRSV-positive. This was the only movement of pigs between regions. At the time of the outbreak, pigs were moved from sites in Voronezh to Lipetsk and Tambov (personal communication, Director of Operations of Infected Swine System, February 2021). Movement of pigs was by commercial trucks with site specific trailers. It was not believed that personnel transported the virus as there were very few personnel that work between regions (personal communication, Director of Operations, Infected Swine System).

### 2.2. Overview of Sites Included and Data Provided

The affected company provided pig movement, diagnostic, and clinical data gathered from a cohort of 30 sites in their swine system from March to November 2021: 2 in Voronezh, 8 in Lipetsk, 13 in Tambov, and 7 in Penza. There were 25 sites with clinically ill pigs that were diagnosed for PRRS using a real time reverse-transcriptase polymerase chain reaction (RT-qPCR) assay and open reading frame (ORF) 5 sequencing in a system that was previously free from PRRSV. Of the 25 sites, 20 had clinical data available, RT-qPCR test results, and 28 viral sequences of the ORF5 from the PRRSV. Four sites had repeat diagnoses of the same clade during this period, the findings from the first diagnosis were used in this analysis. Observations of sites that had two separate diagnoses of different clades (*n* = 4) were included in this analysis. There were 22 observations included in the analysis: 2 in Voronezh, 6 in Lipetsk, 10 in Tambov, and 4 in Penza. One site in Lipetsk was being used as a quarantine station and was transitioning to a sow site at the time of these outbreaks. For this analysis, it is identified as a sow farm. Pig movement data included origin and destination sites and week of movement. Clinical data were not standardized across sites with some reporting specific clinical signs and others just stating there were clinical signs. There was also a concurrent pseudorabies virus outbreak, therefore clinical evaluations were excluded from this study.

### 2.3. Summary of RT-qPCR Diagnostics and Samples

Production staff at the affected site collected samples and shipped them to the Cherkizovo laboratory. Samples were collected from clinically ill and dead pigs, and included either serum, lung tissue, and/or pen-level oral fluid samples. All diagnostics were completed at the Cherkizovo Laboratory, a commercial laboratory that is International Organization for Standardization (ISO) 17025 accredited by the Association of Analytic Centers (Moscow, Russia), which is a member of the International Laboratory Accreditation Cooperation (ILAC) group. The laboratory provides guidelines on proper shipping and the result report contained information on sample collection and integrity. This included the date and location of sample collection, the quality of the package on arrival at the lab, the samples submitted, and the name of the submitter. This analysis used RT-qPCR and sequencing results from 12 lung tissue samples, 7 serum samples, and 3 oral fluid samples. Viral RNA extraction was performed on the samples submitted using a KingFisher Flex^TM^ (Thermo Fisher Scientific, Waltham, MA, USA) using a M-Sorb-OOM kit (Synthol, Russia). Amplification of nucleic acid for PRRSV detection used a RT-qPCR kit produced by AmpliSense (Moscow, Russia) on a Bio-Rad CFX-96 thermocycler (Bio-Rad Laboratories, Hercules, CA, USA). The primer and probe sequences were not publicly available. All procedures followed the kit’s manufacturer’s instructions. Differential diagnostics were run for African and classical swine fever, swine influenza A, pseudorabies virus, *Glaesserella parasuis* (formerly *Haemophilus parasuis*) and *Mycoplasma hyopneumoniae*. No site tested for all the differential diagnoses when testing for PRRSV, and some did not test for any.

### 2.4. Sequence Data Analysis

The Cherkizovo Laboratory used a 3500 Genetic Analyzer (Thermo Fisher Scientific, Waltham, MA, USA) to sequence the ORF5 gene segment from samples with a positive real time PRRSV RT-qPCR with the purpose of differentiating wild type and vaccine strain PRRSV. Primers used for sequencing are considered proprietary and are not available for publication. The 30 sites in the production system submitted 31 cases for sequencing. Those 31 sequencing requests included 54 samples. No site reported more than one clade sequenced at one time even if multiple samples were submitted. Of the 31 sequencing requests, 14 submissions submitted one sample and 17 submitted more than one sample for sequencing. In total 25 sites were positive for PRRSV via RT-qPCR and 18 sites provided 28 sequences for evaluation. These sequences have been submitted to the GenBank database as accession numbers MZ911755–MZ911782. An additional 100 sequences were obtained from GenBank using BLASTn and data cleaning was carried out to remove duplicate sequences (sequences that were 100% identical to at least one other sequence) where the earliest sequence was retained. After this data cleaning step, 91 GenBank sequences were included in the analysis. The GenBank sequences were combined with the 28 outbreak sequences and the Lelystad vaccine sequence was also included in the data, hence a total of 120 sequences were used for subsequent analysis. Multiple sequence alignment was subsequently carried out using the MUSCLE algorithm in ALiView (v 1.26) using default settings [15]. Detection of recombinants was carried out using the recombinant detection program (RDP v 4.1) [16] and no recombinants were detected. Initial tree topology was assessed using MEGA-X 10.1.8 to construct a maximum likelihood phylogenetic tree (using General Time Reversible with gamma distribution (GTR + Γ) model and 100 bootstraps, and the molecular signal assessed using TempEst (v 1.5.3) [17].

We subsequently reconstructed the viral population dynamics using a Bayesian Skygrid coalescent model [18], with a general time-reversible and discrete gamma distribution (GTR + Γ) substitution model [19,20], and an uncorrelated lognormal relaxed clock using Bayesian evolutionary analysis by sampling trees (BEAST) (v1.10.4) [21] on the Extreme Science and Engineering Discovery Environment (XSEDE, accessed on 26 July 2021) on the Cyberinfrastructure for Phylogenetic Research(CIPRES) portal (v 3.2) [22]. We used 500 million Bayesian Markov chain Monte Carlo (MCMC) cycles sampling every 50,000th state to achieve adequate effective sample size. A separate Skygrid coalescent + GTR + Γ model was used to estimate the effective viral population for the two wildtype outbreak clades using 500 million chains sampling every 50,000th. Using Tracer (v 1.7.1) [23], we evaluated model convergence excluding 10% of the maximum clade credibility (MCC) chain as burn-in and ensuring an effective sample size (ESS) of at least 200. A MCC phylogenetic tree was subsequently generated and the ggtree package in R (v 3.1.3.992) was used for visualization [24]. Highest posterior density (HPD) of nodes in the clades to which our query sequences belonged were used to estimate time of divergence of the branches from their ancestors.

### 2.5. Statistical Analysis and Mapping

For diagnostic data, frequencies and percentages were calculated and compared within categories using a Fisher Exact test. The level of significance was set to 0.10 due to low sample sizes. Stata software, version 16.1 IC (College Station, TX, USA) software was used for analysis.

QGIS 3.10 with administrative boundary files from the GADM (www.gadm.org, accessed on 9 December 2020) were used to visualize the spatiotemporal relationships between sites and their infection status (QGIS Development Team, 2021. QGIS Geographic Information System. Open-Source Geospatial Foundation Project. http://qgis.osgeo.org, accessed on 15 January 2021). The infection status was determined by RT-qPCR for detection of PRRSV nucleic acid and through sequencing. The sequences from samples positive for PRRSV nucleic acid on RT-qPCR were classified as infected unknown (not sequenced), infected with a clade 1 variant, infected with a clade 2 variant, infected with a clade 3 variant, or not infected.

## 3. Results

### 3.1. Diagnostic Testing Results and Clinical Presentation

In this case, 25 separate PRRSV diagnosis were made across 20 sites with complete information regarding clinical presentation based on positive RT-qPCR results. Two sites had two wildtype PRRSV variants detected, and two sites had a wildtype and vaccine variant detected during the same period. Two sites had sequencing from the RT-qPCR samples that was not successful in identifying a clade. Clinical signs include erythema of the skin, neurologic signs, respiratory disease, and increased abortion and mortality rates across infected sites. Inconsistent data collection and the presence of pseudorabies virus in the system does not allow for a representative summary of the clinical presentation for PRRSV infection.

### 3.2. Sequencing Results

**Figure 1:**
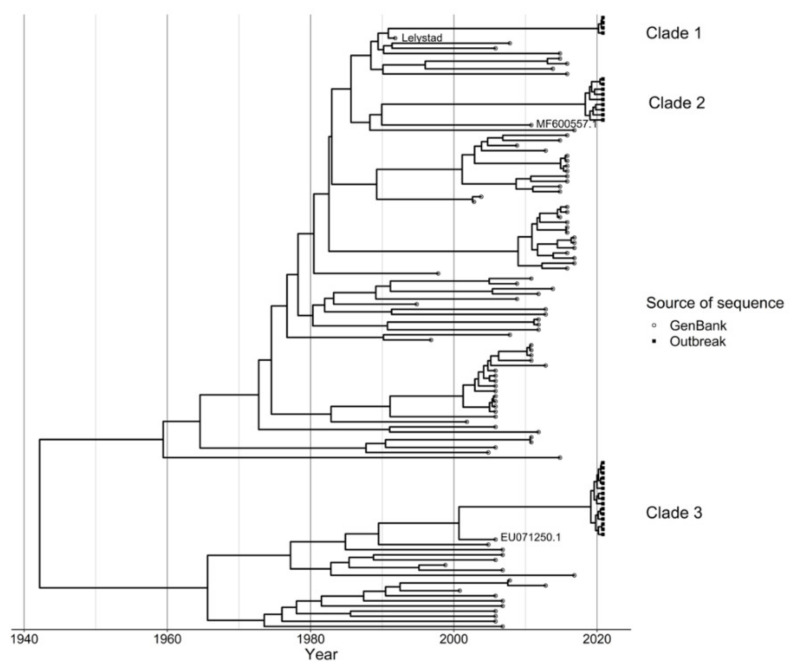
A time-scaled MCC phylogenetic tree of 120 PRRSV type 1 sequences with the tips’ shape distinguishing between outbreak sequences and sequences obtained from GenBank. The outbreak sequences are distinctly marked by square tips and clades are labelled 1–3.

The 28 PRRSV sequences obtained from the PRRSV RT-qPCR positive samples collected from sites involved in the outbreak were clustered into three distinct viral variants defined by clades (Figure 1). Clade-1 was comprised only of vaccine strains, whereas clades-2 and -3 were comprised of wild type viruses. All clade-1 variant were closely related to the Lelystad PRRSV1-1 subtype (Figure 1). The other outbreak viral sequence clusters were each closely related to a different virus. For variants with clade-2 sequences, the closest related sequence was (MF600557.1), which was initially isolated in Poland in 2010. For outbreak PRRSV variants in clade-3, they were closely related to a sequence from a virus that was initially isolated in Russia in 2005 (EU071250.1). Both the MF600557.1 and EU071250.1 sequence were also classified as PRRSV1 subtype 1 variants. The average genetic distance between the outbreak clades-1 to -3 ranged between 4–15%. The genetic distance between, clade-1 and clade-2 was 4.2% (se = 1.9%). Variants with clade-3 sequences were most dissimilar from the other clades with a genetic distance of 15.3% (se = 8.5%) and 15.4% (se = 8.4%) from clade-1 and -2, respectively. The outbreak clades were almost monophyletic, the within clade identity was about 99% for each of the clades, providing about 99% similarity between the sequences in each clade. Specifically, the average within clade distances were 0.0% (se = 0.1%), 0.9% (se = 0.4%), and 0.4% (se= 0.2%) for clades 1, 2 and 3, respectively.

**Figure 2:**
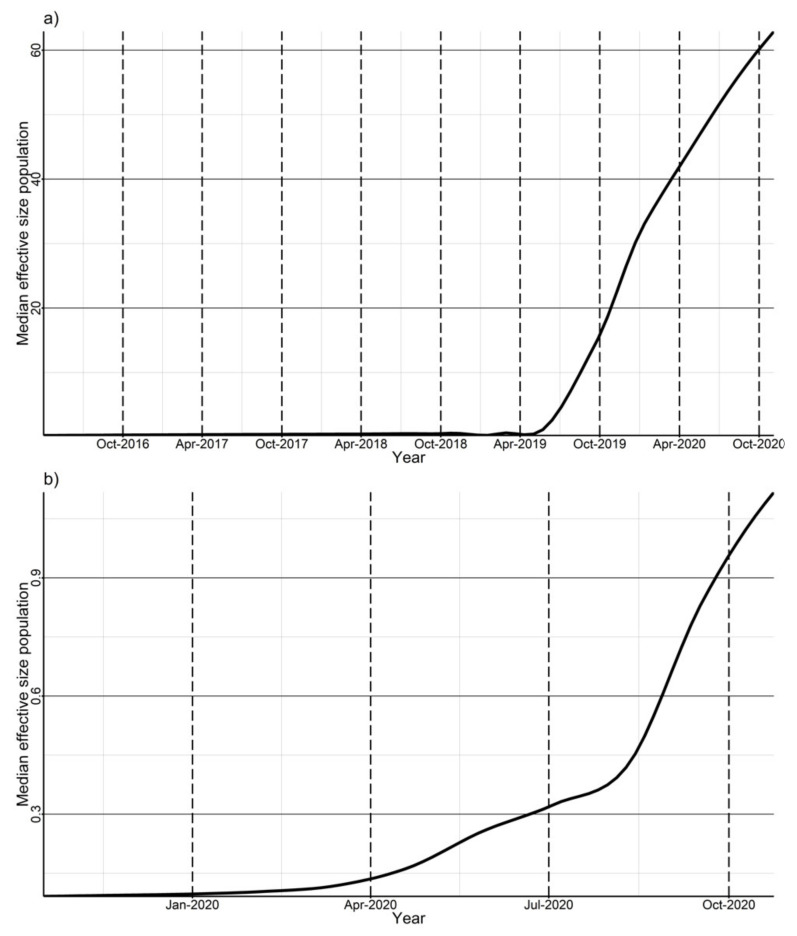
(**a**) SkyGrid plot for wildtype viruses in clades 2. (**b**) SkyGrid plot for wildtype viruses in clades 3. Clade 2 viruses appear to have been circulating in the region in 2019, but the viral population increased considerably from mid-2019. Clade 3 viruses are appeared to have been introduced late 2019 early 2020 and the viral population increased considerably from August 2020.

Time to the most recent common ancestor (TMCRA) for clade 2 was around September 2019, with a 95% highest posterior density (HPD) interval between February 2016 and February 2020. For clade 3, TMCRA was February 2020 with a 95% HPD interval of September 2019 to April 2020. From the Skygrid analysis, we observe that the estimated viral population for both clades started increasing exponentially circa mid-2019 and August 2020 for clade 2 and 3, respectively (Figure 2a,b). SkyGrid analysis to estimate TMRCA and viral population dynamics for clade-1 sequences was not carried out because vaccination entails exposing the pig population to a ‘frozen in time’ virus; the effect was akin to resetting the molecular clock within clade-1 sequences.

### 3.3. Distribution of Clades and Pig Movements That Contributed to Viral Spread

**Figure 3:**
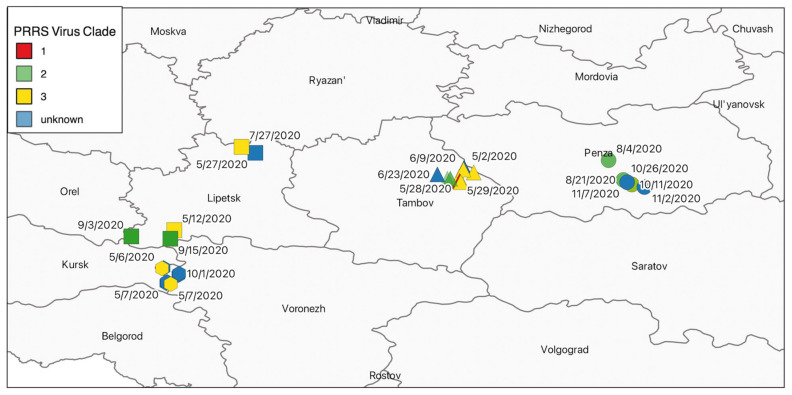
Map of infected pig production sites by region, date of wildtype porcine reproductive and respiratory syndrome virus variants detected, and clade, 2020. (Map created on 27 January 2022. QGIS Development Team, 2021. QGIS Geographic Information System. Open Source Geospatial Foundation Project. http://qgis.osgeo.org, accessed on 15 January 2021).

**Figure 4:**
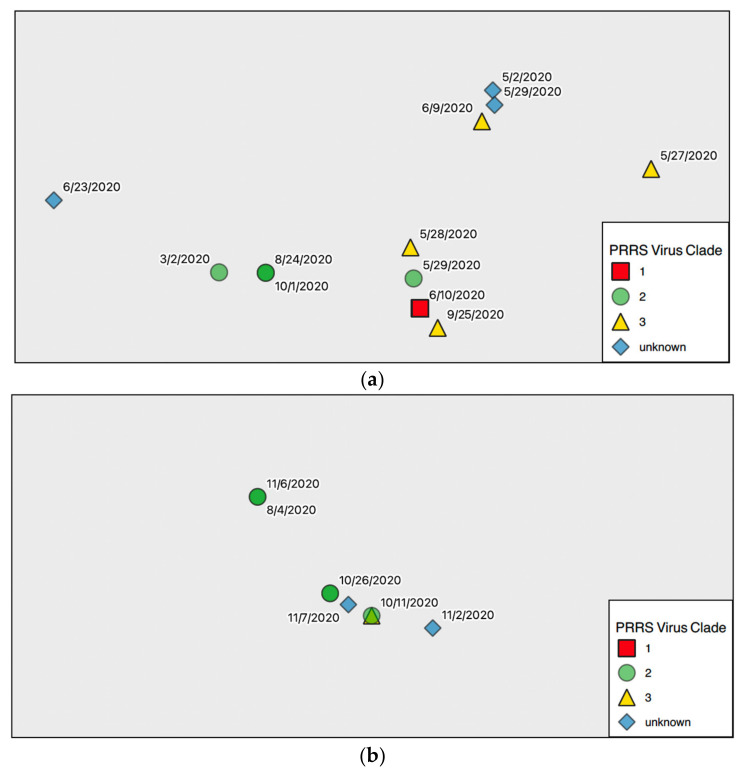
(**a**): Map of the infected pig production site by clade in the Tambov region. They were labeled by clade and date of diagnosis. A few sites were infected more than once and have two dates. (Map created on 2 August 2021. QGIS Development Team, 2021. QGIS Geographic Information System. Open Source Geospatial Foundation Project. http://qgis.osgeo.org, accessed on 15 January 2021). (**b**): Map of the infected pig production site by clade in the Penza region. They were labeled by clade and date of diagnosis. A few sites were infected more than once and have two dates. (Map created on 2 August 2021. QGIS Development Team, 2021. QGIS Geographic Information System. Open Source Geospatial Foundation Project. http://qgis.osgeo.org, accessed on 15 January 2021).

Sites in Voronezh and the northern portion of Lipetsk mostly had viral variants classified as clade-3. The southern portion of Lipetsk, as well as Tambov and Penza had viral variants classified into both clade-2 and -3. All samples that were not sequenced but were RT-qPCR positive for PRRSV nucleic acid were coded as “unknown clades” (See Figure 3). Although the outbreak began in Tambov in March (See Figure 4a), its spread in the system was not evident until May when cases increased in Voronezh, Lipetsk, and Tambov. Affected Voronezh sites provided replacement breeding female pigs to sow farms in the system. Lipetsk’s infected sites were mostly growing pigs (finisher and wean-to-finish) sites, Tambov had a mix of infected sites including sow farms, nurseries and growing pigs, and Penza infected sites were mostly in growing pigs with one sow farm (Figure 4b). The virus was not detected in Penza until July. The system remained positive with outbreaks into the fall, primarily in Penza.

Sites in Tambov and 4 experienced repeat diagnoses of PRRSV over multiple months (See Figure 4a,b).For example, the outbreak began in Tambov in March 2020 when a clade-2 virus was identified. In this region, most sites were infected with PRRSV between May and June, but there were active sites diagnosed with PRRSV into October. Some sites experienced multiple outbreaks with several months in-between diagnoses. For example, one finisher site in Tambov was diagnosed with clade-2 in both August and October. In Penza, sites were first diagnosed in August, and the next diagnoses in those locations occurred in October. More specifically, one finisher site had a clade-3 variant identified in August and a clade-2 variant in October. Another finisher site was diagnosed with a clade-2 variant in both of those months. Since the system widely used PRRSV vaccine which is a clade-1 variant, all system clade-1 variants were excluded from the distribution assessment as they are expected throughout the system.

Inter-regional pig movements were temporally and spatially linked to infection of sites with the clade-3 variant. For example, in April, weeks 14 to 18 of the year, a Voronezh site moved pigs to a site in Lipetsk. Then, a clade-3 variant was identified in Voronezh in week 19, which is the first week of May, and in Lipetsk during week 20, the second week of May. Another example is as follows. During week 19, Voronezh consistently moved pigs from a site that had been infected with a clade-3 variant to a site in Tambov, then the site in Tambov was diagnosed with PRRSV in week 22. There were not any inter-regional movements that were clearly associated sites infected with the clade-2 variant.

There were differences in the distribution of the variant clades across production stages (Table 1). Clade and production stage were significantly associated (*p*-value = 0.04). Clade-3 wildtype variants were found on previous quarantine, gilt development, multiplier, and farrow-to-wean sites (“sow sites”), but clade-2 viruses were not found on sow sites. These sow site detections began in April 2020 in Voronezh. The last farrow-to-wean site diagnosed with a clade-3 variant was in Tambov and was diagnosed in early October. Clade-3 variants were also found on nursery, finisher and wean-to-finish sites (“grower sites”) along with clade-2 variants. Among these grower sites, the first site diagnosed with a clade-3 variant was diagnosed in Lipetsk in May 2021, and the last was in Penza in October 2021. Clade-2 variants were also found in grower sites but not sow sites.

## 4. Discussion

This study described a PRRSV outbreak and the viral spread associated with two wildtype viral variants in a commercial swine production system in Russia. There is confidence in the PRRSV diagnosis as it was not found in the system prior to these diagnoses and was diagnosed in clinically ill animals repeatedly throughout the system, including in sites where pseudorabies was not diagnosed, and pseudorabies vaccine was not used. It was also not possible to confirm the PRRSV diagnosis with animal experiments, but this is a common challenge in production systems. The movement of PRRSV and the introduction of two wildtype variants into the system were critical to assess as biosecurity in swine systems is impacted by two major components: the vulnerability of any one site, and the vulnerability of the contacts between sites. This speaks to the need of sequencing in surveillance, particularly once a disease is circulating in a system, to develop the best control methods given the flow of pigs infected with the given variant.

With increasing accessibility to sequencing and the advancement of analytical models, sequence data are continuously being adopted by production systems for diagnosis and investigation of outbreaks, including the assessment of transmission pathways of pathogens. In swine production, Sanger sequencing is routinely used as it is useful for variant identification and affordable for production systems. In this analysis we observed that PRRS cases reported by 24 farms in this production system were not all caused by the same virus, but instead the wildtype viruses belonged to two different clades. We were able to evaluate the clinical presentations and viral distribution based on clade and trace-back the likely time of emergence of the strains. This analysis also reveals challenges with the use of sequence-based analyses. The repeated introduction of preserved sequences in the system may result in an inaccurate TMRCA estimation, and the genetic diversity within the clade may be masked by the reintroduced isolates through vaccination. Bioinformatics expertise is needed to properly use advanced analyses for these purposes in production systems.

Sequencing of the PRRSV ORF5 does provide only a limited snapshot of viral reassortment. Nonetheless, it revealed that the swine system impacted suffered a severe PRRS outbreak in 2020 caused by wildtype viruses with two distinct sequences. These sequences were primarily related to previously recognized wildtype viral sequences from subtype PRRSV1-1. It was surprising to those managing the system that the clade-1 variant, the most similar to the vaccine strain, caused some clinical illness as well. Severe disease is unusual with PRRSV1-1 variants. Historically, the subtype 1 viruses are typically of lower pathogenicity [25]. Without sequencing, the system would have been unaware that it was struggling with two different outbreaks caused by the same viral genus.

Based on the phylodynamic analysis, the viral strains were estimated to have been circulating in the area at least 1 year before the outbreak, and the effective population of these viruses increased considerably around mid-2019 and August 2020 for the clade 2 and 3 strains, respectively (Figure 2a,b). This increase may have been due to some perturbations in the population causing changes in the dynamics of circulating viruses in the area, subsequently manifesting in the outbreak reported in 2020 in the study production system. Perturbations in the population could be due to animal movements or changes in herd immunity profiles which may be influenced by immunization protocols. Further research comparing the immune response to these “emerging” strains to that conferred by vaccines used in the region may elucidate the observed dynamics of circulating viruses. This analysis would also be enhanced by analysis of sequences circulating among all producers in these regions, but this study is limited to one production system.

In total, eight sites were diagnosed with clade-2 and 15 with clade-3 viruses. There was a difference in the transmission patterns between clade-2 and clade-3 viruses in the system, as sow sites were only infected with clade-3 variants of PRRSV. The number of sites with diagnosed PRRSV infections increased after the emergence of the clade-3 virus in the system in April 2020. Only one finisher site was diagnosed with clade-2 viruses in March 2020, and then in April, a multiplier site was diagnosed with a clade-3 virus. The infection at a multiplier site may have been a result of environmental spread or aerosol. Another company Voronezh was also experiencing a PRRS outbreak from a genetically similar PRRSV variant at that time of the outbreak. A common road that went past the first infected Voronezh site was used by that company to haul infected pigs at the time that site became infected (personal communication, Director of Operations, Infected Swine System).

Another viral transmission route identified was related to the routine pig movements in the system. Throughout the period evaluated, gilt development, multiplier, and farrow-to-wean sites (the sow system) were only impacted by clade-3 viruses, while nursery, finisher, and wean-to-finish sites were impacted by both clade-2 and -3. The dual infection of market pig sites was to be expected. Clade-3 variants were introduced since piglets born on farrow-to-wean sites infected with clade-3 variants moved to nursery and then finisher sites or to wean-to-finish sites and brought infection with them. There was no movement backward to the farrow-to-finish, preventing them from being infected with clade-2 variants. Finisher sites infected with clade-2 variants had contact with nurseries within the region and could infect them. This was likely carried out for fomites (such as vehicles) and personnel. It is not clear how virus spread to Penza, but, again, fomites are likely. Movement between regions is the likely cause, and such large scale of movement is an ongoing risk for the geographic spread of pathogens. The clade-3 movements in this system used a multiplier to repopulate its gilt development sites. As a result, pigs never moved from clade-2 infected sites (nurseries, finisher, and wean-to-finisher) to farrow-to-wean sites. Clade-3 variants were spread within the sow system when gilts from the Voronezh multiplier site were moved to gilt development and farrow-to-wean sites in different regions.

Pig movements were associated spatiotemporally with disease movement. Breeding animals likely had a lesser risk of disease introduction as the backward flow of pigs from nurseries, finisher, or wean-to-finish sites to gilt development sites in a system was limited. This appeared to be protective for the sow sites as they did not acquire clade-2 variants, but nursery sites did not have the same benefit from this pig flow, as they did acquire clade-2 viruses that were originally found at finisher sites. Shared personnel and equipment could also be the route for the infection of nurseries with clade-2 variants. Due to the different levels of biosecurity between production sites, conducting surveillance at nursery and finisher sites as well as farrow-to-wean sites is needed to determine what viruses are present in a system.

The most concerning finding from this study was the introduction of two distinct PRRSV variants, clade-2, and clade-3, within a short period. These variants are closely related to other pathogenic PRRSV that have been circulating for several years and the evidence suggests that these two variants emerged in early 2020. These findings raise concerns regarding the biosecurity of the system. These two isolates were introduced into the system within a three-month window of one another. There was evidence of animal movement after diagnosis in the system and after a farm was known to be positive. Inadequate truck washing or failing to wash the truck at the site where pigs were delivered were also recognized as potential biosecurity breaches. Given the threat of airborne spread of PRRSV (Dee et al., 2005), the presence of backyard production units around the Voronezh region sites could also have contributed to increased disease pressure if there is not adequate biosecurity and pathogen control. Rigorous and continuous biosecurity is needed as the pathogen pressure in this region is high for PRRSV viruses. Air filtration of pig housing can help mitigate some of the disease pressure by reducing transmission into the pig barn [26,27,28]. Pig housing can be retrofitted with air filtration as this has been carried out on farms managed by Pipestone in the United States.

This study used data from a recent outbreak in a commercial swine system that conducted RT-qPCR diagnostics to detect PRRSV nucleic acid as well as sequencing. The data was limited in its suitability to evaluate the distribution of clade-specific isolates as only 28 samples were sequenced and classified. Testing for comorbidities was inconsistent and the methods used to describe clinical signs and mortality rates were not standardized. This precluded us from assessing any clinical differences. Nonetheless numerous areas of improvement were identified. Biosecurity protocols must be continuously assessed and improved. Pig movements should be limited from sites with pending diagnostic results, or pigs should move into quarantine rooms on the receiving site. PRRSV spreads readily in this region as well, and there is some evidence that a nearby system infected this swine system, which may be alleviated by filtration of farms, particularly multiplier and farrow-to-finish locations as a great deal of pig movements begin at these sites. Additionally, understanding the association between vaccination trends, the immune response conferred by the vaccines to emerging strains, and the production impact of those strains could help in the early detection of more problematic strains in the field. Finally, this study reveals the strength of sequencing and molecular epidemiology techniques for commercial swine systems. This capability allowed for recognition of multiple PRRSV variants and to associate variant spread with pig movement between regions. Sequencing and variant identification should be carried out routinely for diseases of concern.

## Figures and Tables

**Table 1:** Distribution of clades by production stage for porcine reproductive and respiratory syndrome diagnoses in a large swine production system, March to November 2020.

	Clade 1	Clade 2	Clade 3	*p*-Values
	# (%)	# (%)	# (%)	α = 0.10
# Sites affected	4 (18.2%)	8 (36.4%)	10 (45.5%)	
Production type ^†^				0.04
Finisher *	2 (50%)	7 (87.5%)	3 (30%)	
Sow farm system *	1 (25%)	0 (0%)	6 (60%)	
Nursery	1 (25%)	1 (12.5%)	1 (10%)	

^#^ Indicates the frequency of occurrence. ^†^ Two finisher sites did not have sequencing results and were excluded. The sow system includes a previous quarantine site that was being converted to a sow site, multiplier, gilt development, and farrow-to-wean sites. Farms were included in the analysis multiple times if they had multiple breaks diagnosed at different time points with different clades. Repeat diagnoses from the same clade used the data from the first diagnosis. * Finisher and sow system sites were statistically different from each other under pair wise comparisons, *p*-value = 0.012.

## Data Availability

Data is not available.
